# Allergy diagnosis from symptoms to molecules, or from molecules to symptoms: a comparative clinical study

**DOI:** 10.1186/s40413-018-0199-y

**Published:** 2018-09-11

**Authors:** N. Mothes-Luksch, G. Jordakieva, L. Hinterhölzl, A. N. Jensen, P. K. Hallmann, M. Kundi, E. Jensen-Jarolim

**Affiliations:** 1 Institute of Pathophysiology and Allergy Research, Center of Pathophysiology, Infectiology and Immunology, Medical University Vienna, Währinger G. 18-20, 1090 Vienna, Austria; 2 AllergyCare, Allergy Diagnosis and Study Center, Vienna, Austria; 3 Department of Dermatology, Division of Immunology, Allergy and Infectious Diseases, Medical University Vienna, Vienna, Austria; 4 Institute of Occupational Medicine, Department of Internal Medicine II, Medical University of Vienna, Vienna, Austria; 5 Center for Public Health, Medical University Vienna, Vienna, Austria; 6 The Interuniversity Messerli Research Institute, University of Veterinary Medicine Vienna, Medical University Vienna, Vienna, Austria

**Keywords:** ISAC microarray, Component-resolved diagnosis, Skin prick test, Allergy diagnosis, Molecular allergens, IgE

## Abstract

**Background:**

Classical allergy diagnostic workup “from symptoms to molecules” comprises 1) clinical investigation, 2) skin prick- and IgE- testing, and recently, 3) molecular allergy testing. We aimed to examine the diagnostic fidelity of the alternative approach “from molecules to symptoms”, which was recently suggested in the EAACI Molecular Allergology User’s Guide, in a retrospective clinical study.

**Methods:**

Records from 202 patients with clinically suspected allergic sensitizations were extracted from files at two sites applying either the “ISAC-first” workup with IgE-testing by immuno-solid phase allergen chip ISAC112 followed by selected skin prick tests (SPT) or the “SPT-first” starting with SPT followed by the microarray test.

**Results:**

In the ISAC-first procedure significantly less SPTs were performed during allergy diagnosis (median 4 vs. 14). By SPT in 19% of patients in the ISAC-first group and in 34% in the SPT-first group additional respiratory allergens (*p* = 0.014) were detected not positive in ISAC microarray. By ISAC microarray test 18% additional sensitizations were found in the ISAC-first, and 32% in SPT-first cohort (*p* = 0.016). For food allergens 13 and 12% additional sensitizations were detected by the microarray not detected by SPT in the two groups (*p* = 0.800). No additional food allergen was found by SPT in the ISAC-first group, while in 6% of the cases in the SPT-first group detected sensitizations were negative in the microarray.

**Discussion:**

The ISAC-first approach followed by (fewer) SPTs meets the demands for a patient’s tailored diagnostic work-up and therefore can be considered equivalent to the conventional way using the skin prick test as first screening tool, followed by IgE diagnosis.

**Conclusions:**

For the diagnostic verification of clinically suspected allergy, the novel concept “from molecules to clinic” offers a reliable diagnostic workup in shorter time. Due to lower skin test numbers it is especially applicable for young children and seniors, in atopic patients, and whenever skin tests get difficult or unreliable.

**Electronic supplementary material:**

The online version of this article (10.1186/s40413-018-0199-y) contains supplementary material, which is available to authorized users.

## Background

Since its detection, specific IgE represents the only diagnostic biomarker for exposure and sensitization in allergy [1] with a predictive value for clinical reactivity in early childhood [2], for asthma [3], and of significance for selecting patients for allergen immunotherapy [4]. Combined with extract-based in vitro and skin prick tests, it reliably correlates with clinical symptoms in respiratory allergies and to a lesser extent in food allergies [5].

Molecular allergy testing, particularly using allergen microarrays, is currently being implemented into daily diagnostic work-up of allergies. The diagnostic allergy field is thus in transition towards molecular-based allergy diagnostics [6] as recently published in the first “users guide for molecular allergy” [7]. In this handbook, besides the classical diagnostic work-up of type I allergy “from symptoms to molecules”, a novel approach “from molecules to clinic” is discussed.

Currently the diagnostic procedure of type I allergy includes the evaluation of patients’ symptoms, followed by screening skin prick tests with a panel of respiratory allergens and/or food allergens and conclusive specific IgE testing in the serum of the patients. In this setting, on the one hand, relevant allergens could be missed, on the other hand, sometimes skin testing is not possible due to inflamed or atopic skin, a matter which is hotly debated [8, 9].

Therefore we aimed to address the question: Is ISAC microarray screening prior to skin testing of equal value as the conventional approach in the diagnosis of type I allergy?

In detail, we addressed whether ISAC microarray screening followed by patient tailored skin prick testing, instead of skin prick screening with standard allergen panels followed by ISAC microarray testing, are comparable settings for the diagnosis of type I allergy. While a number of recent publications compared different diagnostic tools in type I allergy in adults as well as in children [10–13], there are, to the best of our knowledge, no studies comparing head-to-head two groups with the same technology, but using the top-down or bottom-up approach for the diagnosis of type I allergy.

At present the ISAC microarray method is mostly offered to the patient when diagnostic workup has been completed. However, initial allergy screening by multiplex allergen arrays could be meaningful, especially in polysensitized patients [14, 15], to achieve a comprehensive diagnosis in shorter time, reducing the number of patients’ visits, blood withdrawals and skin tests. Considering the low amounts of serum needed, ISAC is favorable in small children. Equally, IgE testing is important when allergy skin tests get less reliable in inflamed, atopic and aged skin [16]. Furthermore, IgE screening followed by fewer SPTs selected according to the clinical phenotype would reduce the patient’s strain but still meet the international standards [17]. There are no studies on the reliability of molecular allergen microarrays as first-line diagnostic instruments in allergic patients compared to allergic patients undergoing conventional approaches. Therefore, we investigated the data of patients who underwent the usual skin prick screening using a panel of 13 inhalant allergens and 7 food allergens followed by ISAC microarray test, and compared to data from patients who were tested for specific IgE by ISAC microarray first and, depending on the results, skin prick tested with selected allergen extracts only.

The present retrospective observational study thus collected evidence whether the method “from molecules to clinic” has a similarly high accuracy as the approach “from clinics to molecules”.

## Methods

### Study design

This is a retrospective observational study analyzing the data of 202 patients with suspected type I allergy, who had been diagnosed with SPT and the ImmunoCAP ISAC112 allergen microarray. Patients from files of two outpatient units were included that fulfilled the inclusion criteria and that were either tested from “molecules to clinic” or from “symptoms to molecules” according to the EAACI Molecular Allergology User’s Guide [7].

### Patients

Patients with suspected allergy to respiratory, skin or food allergens, mean age 36±17 years, were included from files of two study sites that were tested between January and June 2013. Patients with hemophilia or complement deficiencies were excluded from the study. Inclusion criteria were age above 18, symptoms consistent with allergic sensitization and no contraindication for skin prick testing, and no specific allergy treatment. Files were screened consecutively until 101 patients were extracted from each site. The study protocol was registered and approved by the Ethics Committee of the Medical University Vienna (EK 2002/2012).

Two diagnosis principles according to the EAACI Molecular Allergology User’s Guide [7] were compared. Patient cohort “SPT-first” (*n* = 101) was tested from symptoms to molecules: evaluation of history followed by skin prick screening and subsequent detection of specific IgE by ISAC microarray. For skin prick screening a standard allergen panel (see below), or selected allergen extracts, were used independent of clinical symptoms. Cohort “ISAC-first” (n = 101), was tested from molecules to clinic: evaluation of symptoms and history followed by detection of specific IgE by ISAC microarray and subsequent selected skin prick testing depending on IgE profile. To avoid selection bias, data extraction was performed by a student unrelated to the study team.

### ImmunoCAP ISAC112 method

ISAC112 microarray (Thermo Fisher Scientific Inc., Manufacturer: Phadia AB, Uppsala, Sweden) contains 112 allergen molecules spotted in triplicates derived from 51 allergen sources. The assay was performed according to the manufacturer’s description followed by automated fluorescence-detection scanning and analysis of the signals by MIA® software. Briefly, the chips were pre-washed and each microarray reaction site was incubated with 30 μl of serum at room temperature for 120 min. After a washing step the chips were incubated with 30 μl of IgE detection antibody solution at room temperature for 30 min, and washed again before scanning. Results are reported in semi-quantitative ISAC Standardized Units (ISU): below 0.3 (undetectable or very low), 0.3 to < 1 (low), 1 to < 15 (moderate to high) and 15 or higher (very high) according to the manufacturer’s indications.

### Skin prick test

Skin prick tests were performed according to current guidelines [17] using whole extracts (ALK Abello, Hoersholm, Denmark) from 13 respiratory allergens: pollen from alder, birch, hazel, ash, grasses, mugwort, ragweed (ambrosia), buckhorn plantain, HDM1, HDM2, cat, dog and alternaria; and 7 food allergens: hazel nut, peanut, wheat flour, egg, cow milk, soy and cod fish. Patient cohort “ISAC-first” were SPT-tested with selected allergen extracts depending on the results of specific IgE. Oral antihistamines were discontinued two days prior, and oral sympathomimetic treatments at least 12 h before SPT, in the case the patients. If a patient was on oral steroids for more than two weeks, SPT was postponed until three weeks after the steroid therapy had been stopped. One drop of each allergen was placed 2 cm apart on the forearm and then pricked with a lancet. Buffer saline was used as a negative control while histamine acid phosphate (1 mg/ml) was used as a positive control. Grading of skin prick test reaction was done after 15 min by comparison to histamine positive control, as grade 1 (25% of the wheal area induced by histamine), grade 2 (50% of area), 3 (100% of area), 4 (150% of area), and 5 (200% of area).

### Statistical tests

Sample size was determined based on the assumption that 25% will have an SPT or ISAC microarray result for any specific allergen or allergen mix. The study should have 90% power to detect a sensitivity and specificity above 50% at the 5% (one-sided) level of significance. A sample size of 184 was determined assuming half of the tested individuals being positive in SPT. Accounting for lower rates of SPT positives, this number was increased to 200 (finally 202 were extracted). Reanalysis of power with respect to the comparison of the two approaches revealed the following result: for an overall significance level of 5% (two-sided) and a base fraction of 15% the study has a power of 70% to detect a two-fold difference concerning the number of additionally positive allergens by SPT or ISAC microarray. The evaluation of the data of the patients was done in anonymized form with the help of SPSS 22.0 (IBM Corp., USA). A step-wise binary logistic regression was applied to determine predictive allergens in the ISAC microarray for each SPT allergen. For this purpose SPT results of at least grade 1 and ISAC microarray results with ISU > 0.3 were considered positive. Sensitivity and specificity were determined for the detected ISAC microarray allergens with respect to the SPT results, in addition, positive and negative predictive values were computed based on the assumption that the prevalence corresponds to SPT positivity. Clopper-Pearson exact confidence intervals were computed for these percentages. The two cohorts were compared for distributions of numbers of additional positive ISAC microarray or SPT results by Cochran-Armitage trend tests. *p*-values ≤0.05 were considered as significant. For a differential characterization the following additional designations were chosen: *p* ≤ 0.01: highly significant; *p* ≤ 0.001: extremely significant.

## Results

### Allergen sensitization detected by ISAC microarray versus skin prick testing

The cohorts “ISAC-first” (*n* = 101) and “SPT-first” (*n* = 101) did not differ in terms of allergic sensitizations to major allergen sources as determined by IgE testing and/or SPT (Additional file 1: Table S1).

Patients in the SPT-first cohort had a mean age of 36.3 ± 18.2 and were 64% females, in the ISAC-first cohort the mean age was 35.4 ± 16.6, with 46% females. Asthma was diagnosed in 27% of the ISAC-first and 15% of the SPT-first group. Atopic eczema, rhinitis and conjunctivitis could be detected in 15, 82 and 51% of the ISAC-first and 18, 52 and 36% of the SPT-first group. Some patients had also gastrointestinal symptoms, 11% in the ISAC-first group and 7% in the SPT-first group (Additional file 1: Table S1).

### Results for respiratory allergens

**Fig. 1:**
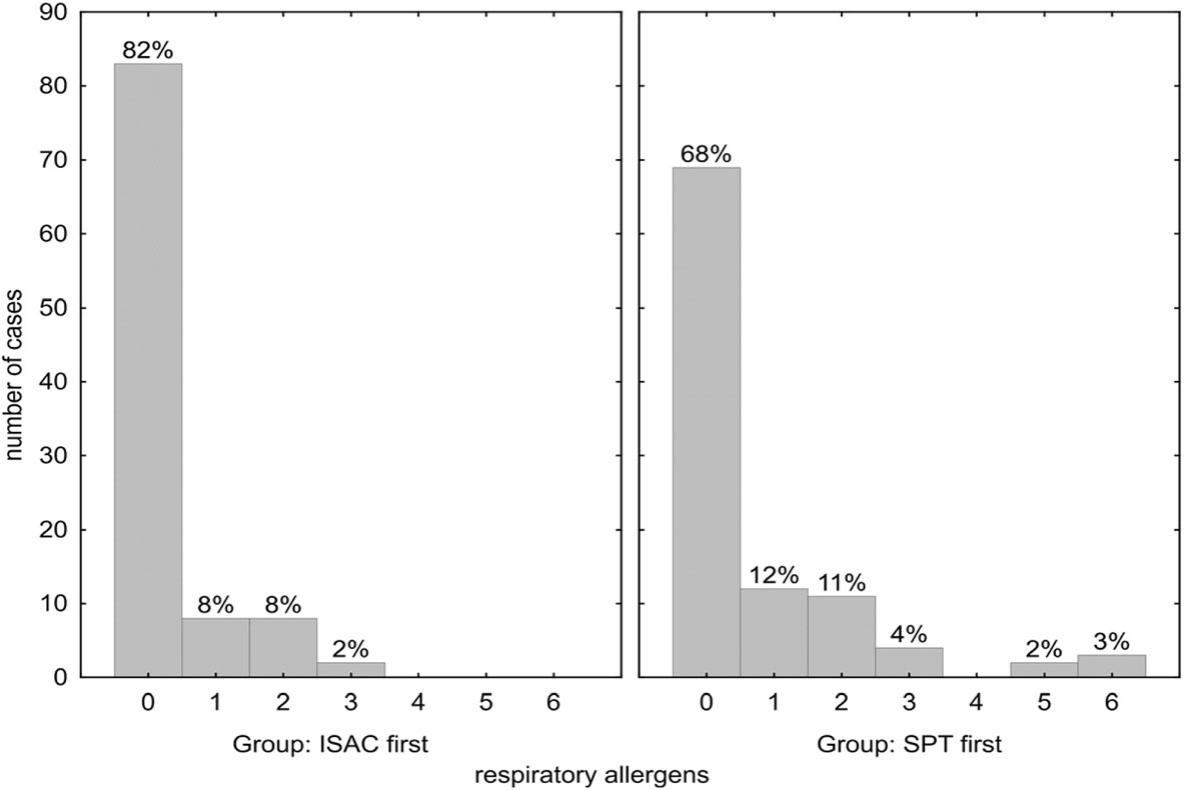
Number of additional positive ISAC results for respiratory allergens not detected in skin prick testing. Within group percentages indicated above the bars. *p* = 0.016 for comparison of ISAC-first vs. SPT-first

In 82% of the ISAC-first group no additional respiratory allergen sensitization was found by the ISAC microarray as compared to SPT (Fig. 1, left panel). In 8% one, in 8% two and in 2% three, hence in 18% additional IgE reactivities were detected without SPT positivity.

In the SPT-first group in only 68% no additional respiratory allergen sensitization was found by ISAC microarray, whereas in 27% up to three, and in 5% five or six additional positive respiratory allergen sensitizations were found (Fig. 1, right panel). The number of detected allergens in the SPT-first group was therefore significantly enhanced by ISAC microarray testing (totally 31% vs. 18%; *p* = 0.017).

**Fig. 2:**
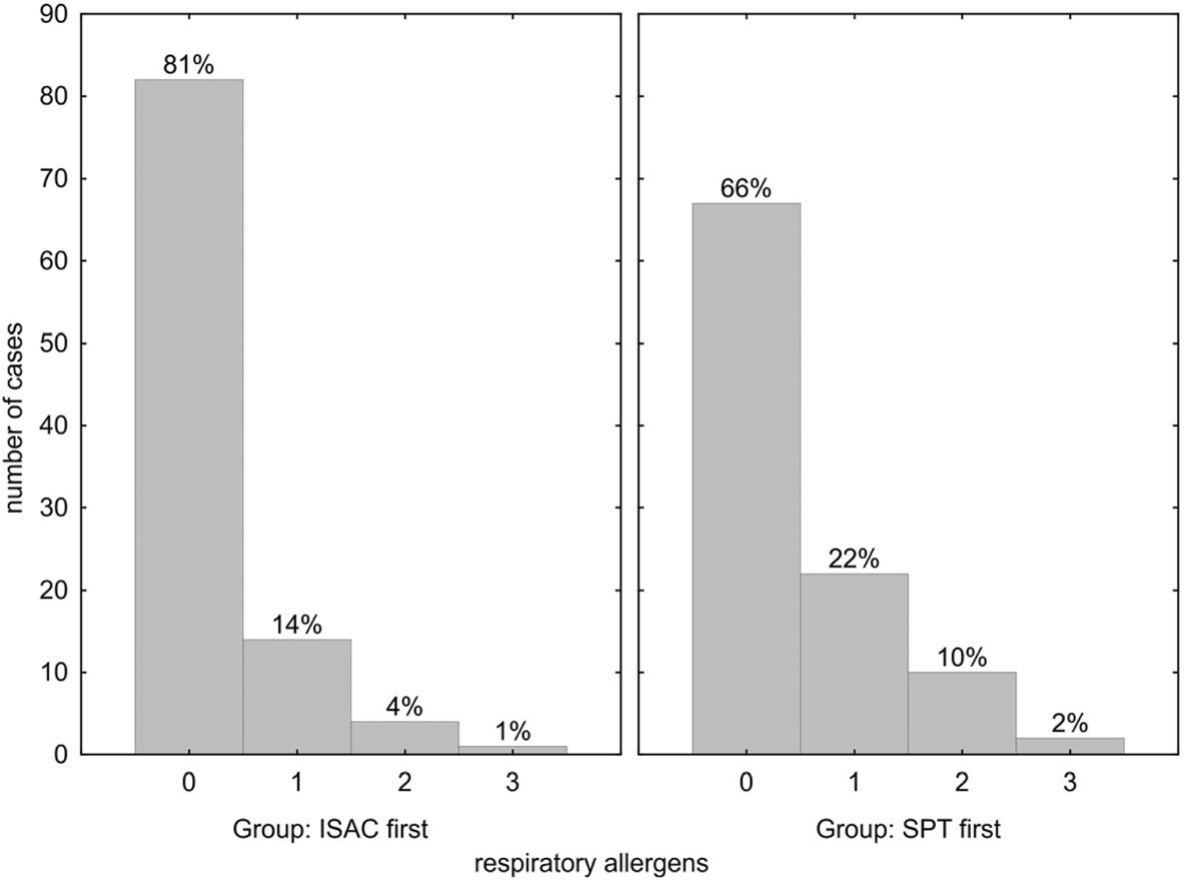
Number of additional positive skin prick results for respiratory allergens not detected by ISAC microarray. Within group percentages indicated above the bars; *p* = 0.014 for comparison of ISAC-first vs. “SPT-first”

Vice versa, in 81% of the ISAC-first group SPT detected no additional respiratory allergen sensitizations (Fig. 2, left panel). In 19% additional respiratory allergen sensitizations were found in SPT, which had been negative in ISAC microarray testing; these were in 14% one, in 4% two and 1% three additional allergen sensitizations in SPT.

In the SPT-first group (Fig. 2, right panel), in 66% no additional allergen sensitizations were diagnosed via SPT, whereas in 22% of this group one, in 10% two and in 2% three additional sensitizations (in total 34%) were found in SPT which were not detected via specific IgE in the ISAC microarray (Fig. 2). Overall, SPT screening in the “SPT-first” patients revealed more respiratory sensitizations not detected by ISAC (totally 34% vs. 19%; *p* = 0.014).

### Sensitivity & specificity analyses, and predictive value of ISAC

Although SPTs were performed in the ISAC first group based on the case histories and ISAC microarray results, sensitivity and specificity were only marginally affected by the different procedures, therefore, results of the combined data are reported. Table 1 shows that the sensitivity and specificity values of the ISAC microarray as well as the positive and negative predictive values of the skin prick test for common respiratory allergens are satisfactory for most allergens. The highest sensitivity was found for Cor a 1.0401 from hazel as predictor for alder (100 [95%CI 81.5–100]) and with a specificity of 77.3 [95%CI 67–7-85.2]; Bet v 1 was a sensitive predictor for hazel (92.9 [95%CI 66.1–99.8]) and birch (97.8 [95%CI 88.2–99.9]), but had a relatively low specificity to predict negative SPTs to birch (77.4 [95%CI 67.6–85.4]) or hazel (65.3 [95%CI 55.2–74.5]. The lowest sensitivity of ISAC microarray was found for mugwort with Art v 1 and Art v 3 not correlating with SPT and only a non-related allergen, Sal k 1, from saltwort pollen, was found predictive with a sensitivity of 20.0 [95%CI 0.5–71.6]) followed by HDM 2 (Der f 2; 75.8 [95%CI 57.7–88.9]) and ambrosia (Art v 1; 61.5 [95%CI 40.6–79.8]).

**Table 1:** Sensitivity and specificity (95% confidence intervals) of ISAC allergens for prediction of skin prick test (SPT) results and of SPT results for prediction of ISAC112 results are shown

		ISAC with respect to SPT		SPT with respect to ISAC	
SPT	ISAC112 allergen	Sensitivity (95% CI)	Specificity (95% CI)	Specificity (95% CI)	Sensitivity (95% CI)
Alder	Cor a 1.0401	100 (81.5–100)	77.3 (67.7–85.2)	45.0 (29.3–61.5)	100 (95.2–100)
Birch	Bet v 1	97.8 (88.2–99.9)	77.4 (67.6–85.4)	67.7 (54.9–78.8)	98.6 (92.6–100)
Hazel	Bet v 1	92.9 (66.1–99.8)	65.3 (55.2–74.5)	27.1 (15.3–41.8)	98.5 (92.0–100)
Ash Tree	Ole e 1	86.2 (68.3–96.1)	81.6 (72.5–88.7)	58.1 (42.1–73.0)	95.2 (88.3–98.7)
Grass	Phl p 1	92.3 (83.0–97.5)	75.9 (66.7–83.6)	69.8 (58.9–79.2)	94.3 (87.1–98.1)
Ambrosia	Art v 1	61.5 (40.6–79.8)	90.0 (82.4–95.1)	61.5 (40.6–79.8)	90.0 (82.4–95.1)
Buckhorn	Phl p 5	87.5 (61.7–98.4)	78.4 (68.8–86.1)	40.0 (23.9–57.9)	97.4 (91.0–99.7)
HDM 1	Der f 2 & Phl p 5	85.7 (69.7–95.2)	73.5 (63.9–81.8)	52.6 (39.0–66.0)	93.8 (86.0–97.9)
HDM 2	Der f 2	75.8 (57.7–88.9)	91.4 (84.4–96.0)	73.5 (55.6–87.1)	92.3 (85.4–96.6)
Cat	Fel d 1	84.4 (67.2–94.7)	75.0 (65.7–82.8)	50.0 (36.1–63.9)	94.2 (87.0–98.1)
Dog	Can f 1 & Ole e 1	85.7 (57.2–98.2)	70.0 (60.5–78.4)	26.7 (14.6–41.9)	97.5 (91.2–97)
Alternaria	Alt a 1 & Can f 1	100 (69.2–100)	82.6 (74.1–89.2)	34.5 (17.9–54.3)	100 (96.0–100)
Mugwort	Sal k 1	20.0 (0.5–71.6)	100 (96.7–100)	61.5 (40.6–79.8)	90.0 (82.4–95.1)

Negative IgE results in ISAC microarray for the 13 respiratory allergens are highly predictive for negative SPT reactivity, while the positive predictive values exceed 50% in only 8 of the 13 respiratory allergens routinely tested in SPT (Table 1). Among the positive predictors most were very plausible, such as Bet v 1 for SPT reactivity to birch, less to hazel; Cor a 1 had an intermediate predictive value for alder; Phl p 1 for grasses; Art v 1 for Ambrosia and Ole e 1 was a marker for ash tree. We observed several unexpected associations between independent allergens (data not shown).

### Results for food allergens

**Fig. 3:**
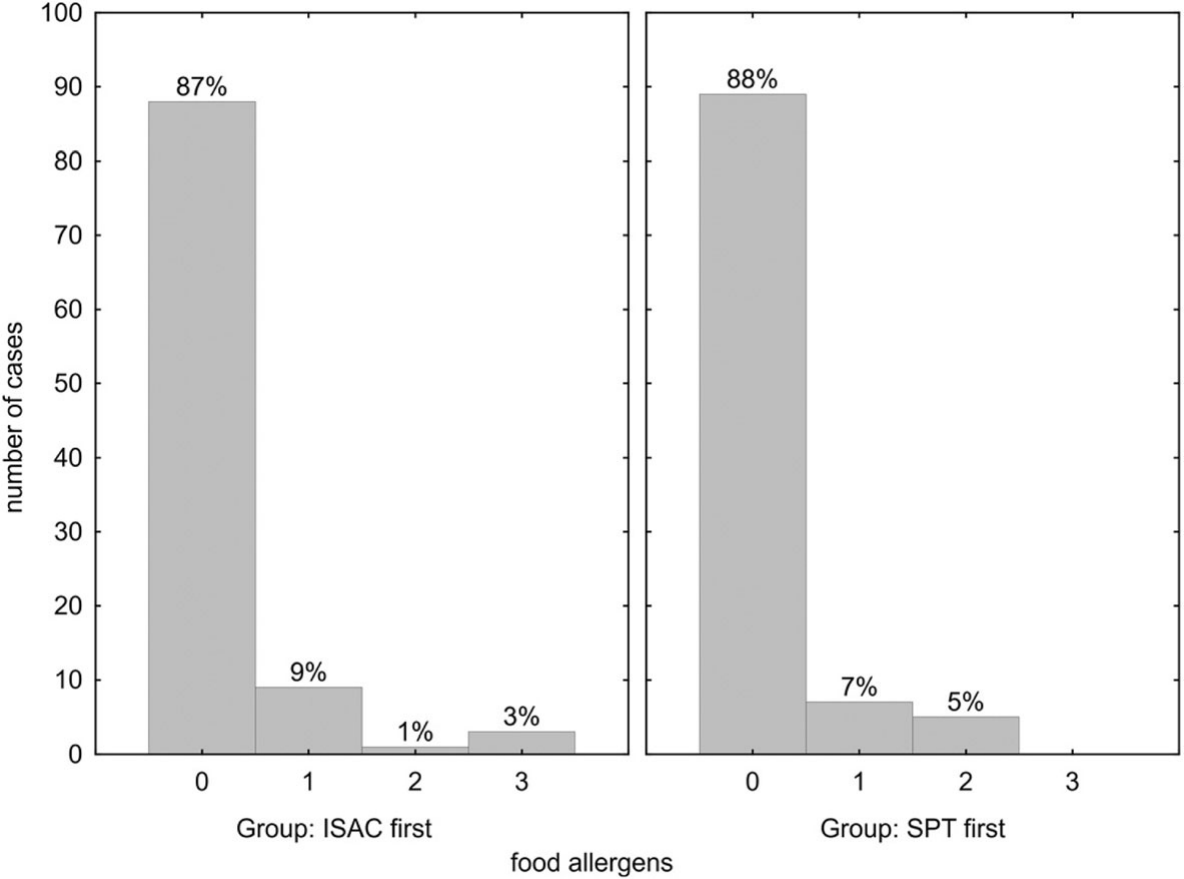
Number of additional positive ISAC microarray results for food allergens not detected by skin prick test. Within group percentages indicated above the bars; *p* = 0.800 for comparison of ISAC-first vs. SPT-first

The ISAC microarray detected no additional food allergen sensitization in 87% of the ISAC-first (Fig. 3, left panel), and in 88% of the SPT-first group (Fig. 3, right panel). In both cohorts in totally 14% of cases IgE to up to 2–3 additional food allergens were detected by ISAC microarray.

Vice versa, SPT in the ISAC-first group detected no additional sensitizations to food allergens, while in the SPT-first group two cases each with positive SPT but negative IgE results in ISAC to wheat flour, hazelnut, and peanut were found (data not shown).

### Results for all allergen categories

**Fig. 4:**
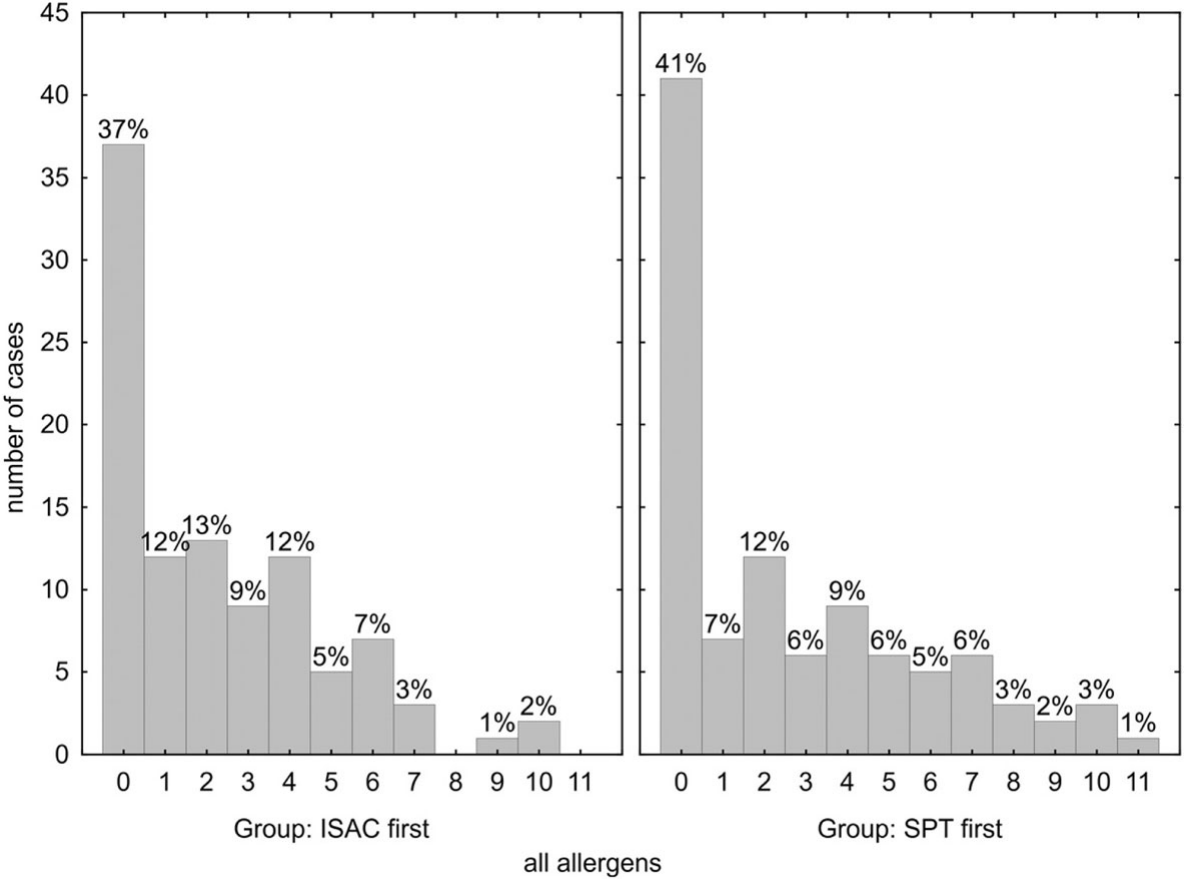
Number of additional positive ISAC microarray results for all allergens not detected/or not done in skin prick testing. *p* = 0.668 for comparison of ISAC-first vs. SPT-first diagnostic strategies

Overall in 37% of the ISAC-first (Fig. 4, left panel), and in 41% of SPT-first group (Fig. 4, right panel), no additional sensitization could be found by the ISAC microarray for allergens not detected or not tested in SPT. The average number of additional positive information achieved with ISAC did not differ significantly between the ISAC-first (mean allergen number 3.6 ± 2.2) and in the SPT-first diagnostic procedures (4.5 ± 2.7) (*p* = 0.044).

The allergens that in addition to SPT were found via IgE by the ISAC microarray in the ISAC-first (Fig. 4, left panel) and SPT-first group (Fig. 4, right panel), could predominantly be classified as “grass-” (25 and 34%), or “birch-related” (19 and 29%), and “HDM” (17 and 16%) allergens, respectively (Table 2). Those three predominant allergen classes also showed the highest concurrency with other examined allergen groups, such as animal epithelia, “high-risk” food allergens, and insect venom allergens as well as with single allergens such as kiwi, soy and latex (Table 2). Concurrent grass pollen sensitizations were more common in subjects with birch- (10 and 19%), HDM- (7 and 12%) and animal epithelia- (3 and 10%) related allergies in the ISAC-first and SPT-first group, respectively. Concurrent HDM allergen sensitizations were more common in subjects with birch-related (9 and 8%) allergies in the ISAC-first and SPT-first group, respectively. In the SPT-first group, latex allergen sensitization was more common in subjects with grass pollen (5%), birch-related (5%) and soy (4%) allergies. In the ISAC-first group, no latex allergen sensitization was found.

**Table 2:**
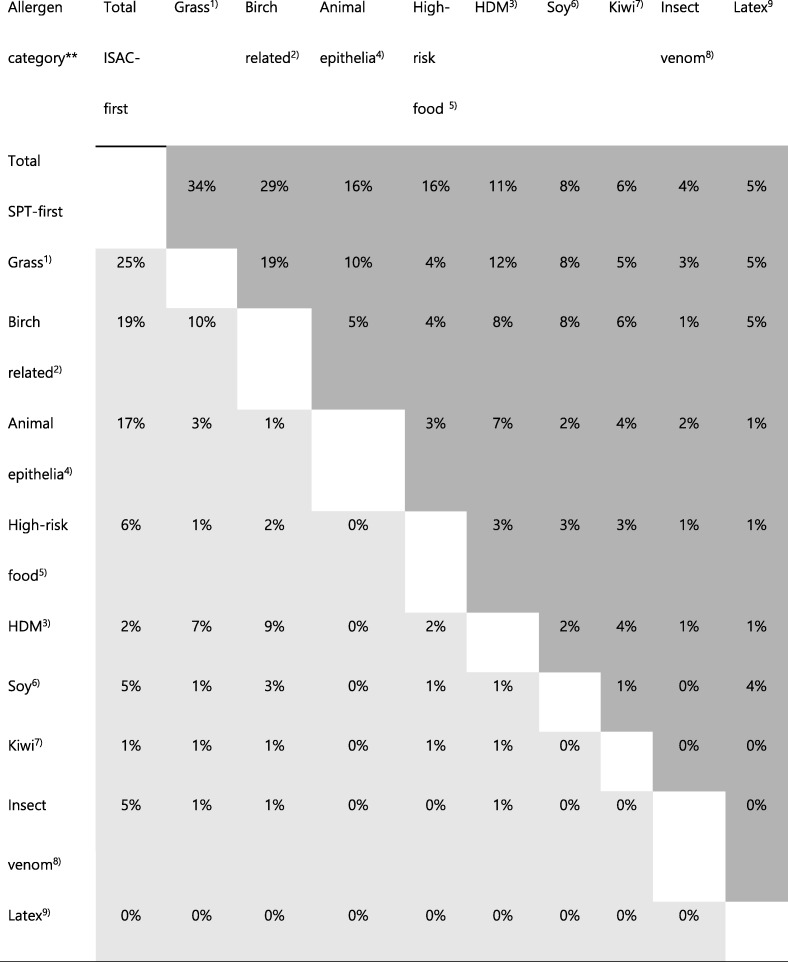
Concurrency^a^ of allergens with positive IgE reactivity in ISAC microarray, but negative in skin prick test

^a^ Concurrent allergen classes for the ISAC-first (light grey) and SPT-first (dark grey) patient groups in relation to total case numbers in percent (%) of the respective cohort

^b^ List of allergens in the different categories:

^c^ Cyn d 1, Phl p 1, Phl p 2, Phl p 4, Phl p 6, Phl p 7, Phl p 11, Phl p 12, Pla l 1

^d^ Aln g 1, Cor a 1.0101, Mal d 1, Ara h 8, Pru p 1, Bet v 2, Bet v 4

^e^ Der f 1, Der p 1, Der p 2, Der p 10

^f^ Equ c 1, Fel d 2, Fel d 4, Mus m 1, Can f 2, Can f 3, Can f 5

^g^ Ara h 1, Ara h 2, Ara h 6, Ara h 9, Pru p 3, Pen m 1, Pen m 2, Gal d 3, Gad c 1, Tri a 14, Cor a 8, Cor a 9, Bos d 6, Gly m 5, Gly m 6, Ses i 1

^h^ Gly m 4

^i^ Act d 1

^j^ Ves v 5, Api m 1, Api m 4

^k^ Hev b 8

### Number of skin prick tests performed

**Fig. 5:**
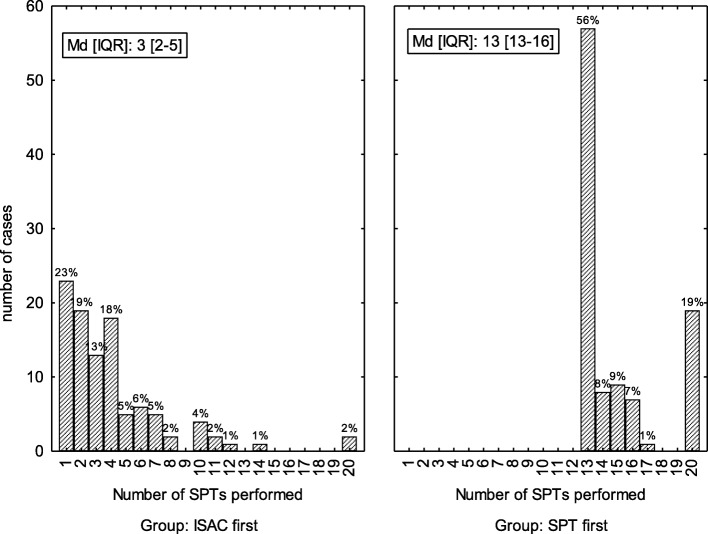
Distribution of the number of skin prick tests performed in the ISAC-first and SPT-first group (x-axis) and number of cases (y-axis). Median (Md) and interquartile range (IQR) are shown in panels

On average 10 SPTs less were sufficient for final diagnosis in the ISAC-first group who after clinical examination were screened by ISAC microarray IgE testing, as compared to the SPT-first group who were routinely SPT screened by a standard panel of 20 allergen extracts (Fig. 5). In the ISAC-first group on average 4 SPTs were performed based on ISAC microarray results and case histories (Fig. 5, left panel). In contrast, in the SPT-first group, a minimum of 13 SPTs were documented per person and 19% had SPTs for all 20 allergens (including 7 food allergens: hazel nut, peanut, wheat flour, egg, cow milk, soy, and cod fish) (Fig. 5, right).

## Discussion

It is generally accepted that molecular allergy diagnosis improves risk evaluation, sorts out genuine from cross-reactive sensitizations, and improves the overall predictive value of the diagnostic results as well as the accuracy of the resulting allergen immunotherapy. It is a tool to monitor allergen exposure [18], for instance, during life time [19], or dependent on residential settings [20, 21], for IgE and IgG [22]. Due to its precise results it may be used to evaluate changes in exposure due to landscape design [23]. Molecular allergy diagnosis therefore may be classified as a method of precision medicine [24]: it improves diagnostic accuracy in polysensitized respiratory allergies [14, 25] where it may also reveal regional differences [21] in food allergies [26, 27], anaphylaxis [4], and atopic dermatitis [28, 29], especially in regards to pollen [30] and house dust mite allergy diagnosis [31]. Although whole allergen extracts are used for SPT while ISAC uses components, results from the ISAC microarray test correspond well with results from other serum tests for specific IgE [32, 33] and with skin prick test (SPT), and recently showed robust performance in a multicenter study [34].

Still, allergy diagnosis is different in daily practice: after evaluating patient’s history and clinical symptoms, usually skin prick screening with 20 up to 100 allergen extracts is performed, followed by serological IgE determination. The WAO-ARIA-GA2LEN consensus document was the first opening up of a diagnostic avenue towards molecular allergy diagnosis, which since then, may replace other IgE tests when performed by the experienced expert [6]. The consequent next milestone was the EAACI’s users’ guide in molecular immunology [7], stating that two diagnostic strategies may be followed in allergy, either “from clinics to molecules” or “from molecules to clinic”. In the present study we intended to investigate whether indeed both strategies are similarly accurate. Two patient cohorts were compared, the ISAC-first cohort after evaluation of symptoms and history was diagnosed by ISAC microarray followed by selected skin prick tests, while the other cohort was first skin prick screened with standard extracts followed by ISAC microarray testing.

Our results are supportive for the concept “from molecules to clinic”, especially using multiplexing IgE detection systems. This technology offering 112 or more allergens [35] for simultaneous testing for specific IgE, indeed could be placed in the beginning of each diagnostic procedure, just after recording the medical history in patients with suspected type I allergy.

Taken together, the diagnostic accuracy of both screening procedures (SPT-first or ISAC-first) was comparable and rendered confident diagnosis, with a few notions: 1.) Significantly more allergens were detected by ISAC112 in the SPT-first group (*p* = 0.017) (Fig. 1), which may be due to the large number of negative results often found in SPTs that would not be encountered by a tailored SPT strategy. 2.) Vice versa, the SPT-first procedure revealed more respiratory sensitizations not detected by ISAC112 (*p* = 0.014) (Fig. 2), due to the higher likelihood to detect positive skin reactions the more SPTs are performed, many of which would be due to cross-reactivity. 3.) In both cohorts a similar number of additional food allergens were detected by ISAC112, but not by SPT in the ISAC-first group (Fig. 3), meaning that ISAC microarray very well covered the required allergen spectrum. On the other hand, SPTs for food allergens were performed less frequently based on patients’ reported sensitivities, which could also explain why there was no difference with respect to additional allergens found by the ISAC microarray between the two groups. 4.) Most importantly, a significantly higher number of SPTs were performed in the SPT-first cohort (a procedure largely resembling current practice) to achieve diagnosis than in the ISAC-first strategy (Fig. 5).

The sensitivity and specificity of the ISAC microarray testing in our study (Table 1) was overall comparable to previous studies [28], and showed a strong correlation with single plex tests including IgE and SPT [13, 36], specifically for respiratory allergens [30, 31], but in fact varied slightly from allergen to allergen. This was also observed in a recent study, suggesting to ultimately approve diagnosis by more sensitive single plex tests, for instance, in cases of anaphylaxis [11]. In our study the highest sensitivities were achieved with the PR10 family molecules Cor a 1 and Bet v 1, followed by Phl p 1. These sensitivities were complemented by good specificities, like in the case of Bet v 1 which showed a 97.8% sensitivity and a 77.4% specificity to birch, followed by a 65.3% specificity to hazel. This could also be observed for Phl p 1 with a sensitivity of 92.3% and a specificity of 75.9%. The mugwort allergen Art v 1, a good predictor for ragweed, showed 90% specificity, and 61.5% sensitivity. Indeed, Art v 1 has a homology to the so called “ragweed homologue of Art v 1 precursor”, varying between 64 to 50% amino acid homologies (from BLAST), without significant similarity with major *Ambrosia* allergen Amb a 1. We therefore hypothesize that the mugwort allergen extract used for SPT contained cross-reacting components that do not seem to be significant for the identification of a genuine sensitization. For *Alternaria* only the genuine allergen Alt a 1 was a predictor of sensitization, with 80% specificity and 100% sensitivity. As expected, Ole e 1 was a good predictor of ash tree (86.2% sensitivity, 81.8% specificity).

In terms of predictive value (Table 1), the binary logistic regression analysis supported already known clinical cross-reactivities among related allergen sources, but also revealed unexpected relations between marker molecules, however, of lower predictive value (data not shown).

Although care has been taken to assess the two procedures as they are performed in everyday clinical practice, there are some limitations that are related to the differences in the two patient groups. There were more males in the "ISAC first" group and there were also some differences in clinical features that could have affected the pattern of sensitizations. Hence, the difference in the numbers of additional allergens detected by SPT or ISAC microarray could have been overestimated; however, the conclusion of an at least equal fidelity of the two clinical work-up procedures seems unaffected by these differences.

With all of these observations taken together, the ISAC-first approach followed by (fewer) SPTs meets the demands for a patient’s tailored diagnostic work-up and therefore can be considered equivalent to the conventional way using the skin prick test as first screening tool, followed by IgE diagnosis.

## Conclusions

Summing up, the concept “from molecules to clinic” has several practical advantages. Based on our data we propose that ISAC microarray testing can be applied as a primary screening approach, preferably i) in settings where skin test results are unreliable, as in acute or chronically inflamed skin including atopic dermatitis [4, 10, 28, 29, 32], ii) or in elderly patients where mast cell numbers are reduced in the skin [16, 37, 38]; iii) when skin tests are not applicable due to inevitable medication interfering with histamine release; iv) in small children when the skin area is limited, and the strain of skin prick testing is higher than in adults; v) in patients who previously experienced anaphylactic events or with suspected complex polysensitization. In particular, in complex cases the number of patient’s visits to get a correctly diagnosed type I allergy can be reduced with the comprehensive ISAC microarray diagnosis. Due to the higher accuracy the right allergen immunotherapy also can be defined in shorter time resulting in an improvement of the overall patient management.

## Additional file

Additional file 1: **Table S1.** Baseline characteristics of patients in the ISAC-first and SPT-first groups. (DOCX 48 kb)

## Abbreviations

h: hour
HDM 1: House dust mite 1 (Dermatophagoides pteronyssinus)
HDM 2: House dust mite 2 (Dermatophagoides farinae)
IgE: Immunoglobulin E
ISAC: Immuno-solid phase allergen chip
min: minutes
RT: Room temperature
SPT: Skin prick test
