# Supplementary material for: Allergy diagnosis from symptoms to molecules, or from molecules to symptoms: a comparative clinical study
Source: World Allergy Organ J. 2018 Sep 11;11(1):22. doi: 10.1186/s40413-018-0199-y (PMC6131881; doi:10.1186/s40413-018-0199-y)
Supplement: Supplementary file 1 — Table S1. Baseline characteristics of patients in the ISAC-first and SPT-first groups. (DOCX 48 kb) [file 40413_2018_199_MOESM1_ESM.docx]

**Table S1.** Baseline characteristics of patients in the ISAC-first and SPT-first groups

|  | ISAC-first | SPT-first | p-value |
| --- | --- | --- | --- |
| females (%) | 46% | 64% | 0.011 |
| age (mean±SD) | 36.3±18.2 | 35.4±16.6 | 0.728 |
| asthma | 27% | 15% | 0.056 |
| atopic eczema | 15% | 18% | 0,704 |
| rhinitis | 82% | 52% | <0.001 |
| conjunctivitis | 51% | 36% | 0.033 |
| gastrointestinal symptoms | 11% | 7% | 0.460 |
